# An Educational Problem

**Published:** 1902-08-09

**Authors:** 


### An Educational Problem.

If, as seems possible, one effect of the Education Bill should be to give to those responsible for the public health a much more definite say in the management of the elementary schools than they have hitherto possessed, some very interesting problems will come up for solution. One of these will certainly be that arising from the fact that compulsory education, as meted out to the people of this country, has signally failed to secure reasonable protection from infection to the children who are compelled to go to school. Even in regard to vermin, which we may take as the coarsest type of infection, one result of the present system has been to impress both children and parents with the hopelessness of all efforts to keep clean. Children of a station in which, in days gone by, lousiness would have been regarded as an abomination and ringworm as a disgrace, have come to look upon these things as inevitable, while mothers have too often, it is to be feared, been driven to give up the unequal fight against an all-pervading dirtiness—a tide of lousiness and ringworm, the ever present source of which in "that school" neutralises their every effort to bring up their children in habits of nicety. The extent to which this evil prevails may be gathered from a recent report by Dr. J. M. Martin, the medical officer of health for Stroud Urban and Rural Districts, wherein he stated that the result of the medical examination of the whole number of scholars in a certain school was as follows :—Two children were desquamating after scarlet fever, ten had impetigo contagiosa, one had scabies, and ninety-two had pediculi in their heads, *i.e.* 43 per cent. of the children were suffering from some contagious malady. We need have no hesitation in agreeing with Dr. Martin when he says that "it is monstrous that children who are clean should be compelled to attend school and so to associate with those who are infected with lice, impetigo contagiosa, scabies, etc.," and we may add that the same may be said in regard to ringworm, the prevalence of which already threatens to bring education to a standstill in some towns.

The considerations which force themselves upon the mind whenever it is turned towards this miserable subject of verminous children have led some people to the conclusion that much of the shibboleth which now passes current among sanitarians in regard to the prevention of infection has but slight relation with the actualities of life. Surely it is idle, they say, to talk about avoiding invisible microbes when we have not yet succeeded in keeping ourselves free from fleas ; surely all this talk of preventing disease by destroying infinitely minute bacilli is beside the mark at a time of day when the comparative gross infection of ringworm is allowed to grow under our very eyes, and when the absolutely massive infection of pediculosis is permitted to go on spreading among children who are under the control of a great Government department.

We look at the problem, however, from quite the other side. The possibility of controlling the spread of disease by interfering with the passage of infection from case to case is so obvious, and the instances in which epidemics have been checked by breaking even one single but essential link in the chain of events by which germs are disseminated are so striking that, instead of despairing of being able to control infection by invisible microbes, we hold that the very fact that diseases which depend upon such microbes are being controlled is proof that all is not being done which ought to be done in checking the spread of that very visible living dirt which is allowed to infect so many of the children attending public rate-supported elementary schools.

No one can now hesitate to admit that the elementary schools of this country are the great central *entrepôts*, the exchanges at which diseases of diverse kinds are distributed from house to house and from street to street, or that one of the most hopeful means at our disposal of diminishing child mortality lies in the sanitary control of these great emporiums of infection. Hitherto the schools have been practically independent. The sanitary authorities—public bodies appointed by law to deal with questions of public health—have had no say in the management of those foci of infection by which the public health has been most threatened ; their officers have been debarred admission to the schools, and prevented conducting any effective medical examination of the children ; and, in fact, the link between the sanitary and the educational authorities has been practically confined to the notification of certain infectious diseases, and to the power possessed by the health officers to close the schools in case of epidemics. All this must be altered before long. If we compel children to go to school we must hold ourselves responsible that they shall not suffer in consequence, and for this purpose all sanitary authorities will be agreed that the first steps must be frequent medical inspection of all children and rigid exclusion of those who are capable of conveying infection to others.

